# Migration pattern of hepatitis A virus genotype IA in North-Central Tunisia

**DOI:** 10.1186/s12985-015-0249-9

**Published:** 2015-02-08

**Authors:** Abir Beji-Hamza, Stefania Taffon, Salma Mhalla, Alessandra Lo Presti, Michele Equestre, Paola Chionne, Elisabetta Madonna, Eleonora Cella, Roberto Bruni, Massimo Ciccozzi, Mahjoub Aouni, Anna Rita Ciccaglione

**Affiliations:** Laboratory of Infectious Diseases and Biological Agents, Faculty of Pharmacy, University of Monastir, 5000 Monastir, Tunisia; Viral Hepatitis Unit, Department of Infectious, Parasitic and Immune-Mediated Diseases, Istituto Superiore di Sanità, V.le Regina Elena, 299, 00161 Rome, Italy; Laboratory of Microbiology, CHU Fattouma Bourguiba, Monastir, Tunisia; Epidemiology Unit, Department of Infectious, Parasitic and Immune-Mediated Diseases, Istituto Superiore di Sanità, Rome, Italy; Department of Cell Biology and Neurosciences, Istituto Superiore di Sanità, Rome, Italy; University Biomedical Campus, Rome, Italy

**Keywords:** HAV, Sequencing, Phylogenetic analysis, Viral gene flow

## Abstract

**Background:**

Hepatitis A virus (HAV) epidemiology in Tunisia has changed from high to intermediate endemicity in the last decades. However, several outbreaks continue to occur. The last reported sequences from Tunisian HAV strains date back to 2006. In order to provide an updated overview of the strains currently circulating in Tunisia, a large-scale molecular analysis of samples from hepatitis A cases was performed, the first in Tunisia.

**Results:**

Biological samples were collected from patients with laboratory confirmed hepatitis A: 145 sera samples in Tunis, Monastir, Sousse and Kairouan from 2008 to 2013 and 45 stool samples in Mahdia in 2009. HAV isolates were characterised by nested RT-PCR (VP1/2A region) and sequencing. The sequences finally obtained from 81 samples showed 78 genotype IA and 3 genotype IB isolates.

A Tunisian genotype IA sequence dataset, including both the 78 newly obtained IA sequences and 51 sequences retrieved from GenBank, was used for phylogenetic investigation, including analysis of migration pattern among six towns. Virus gene flow from Sfax and Monastir was directed to all other towns; in contrast, the gene flows from Sousse, Tunis, Mahdia and Kairouan were directed to three, two, one and no towns, respectively.

**Conclusions:**

Several different HAV strains co-circulate in Tunisia, but the predominant genotype still continues to be IA (78/81, 96% isolates). A complex gene flow (migration) of HAV genotype IA was observed, with Sfax and Monastir showing gene flows to all other investigated towns. This approach coupled to a wider sampling can prove useful to investigate the factors underlying the spread of HAV in Tunisia and, thus, to implement appropriate preventing measures.

## Background

*Hepatitis A virus* (HAV), a member of the family *Picornaviridae*, genus *Hepatovirus*, is the major cause of acute hepatitis throughout the world and causes substantial morbidity in both developed and developing countries [1].

HAV is mainly transmitted by the faecal-oral route. HAV can survive for long in water and numerous epidemics have been observed following consumption of contaminated drinking water, food produce and shellfish [2-10]. The true incidence of hepatitis A is often underestimated because of under-reporting as a result of its widely asymptomatic and milder forms of infection; thus, the epidemiologic pattern is indicated primarily by its seroprevalence.

The epidemiology of HAV is highly correlated with level of hygiene and age. In developing countries, poor sanitary and hygienic conditions, low economic status, high crowding and inadequate water treatment contribute to a high endemicity pattern; the majority of children acquires infection (most often asymptomatic) during early childhood [11,12]. Thus, in these countries overt forms of hepatitis A are relatively rare and severe forms are exceptional [13,14]. The epidemiologic pattern of hepatitis A infection is currently changing in many developing countries where socio-economic conditions are improving: hepatitis A affects the population at a later age, leading to an increased risk of symptomatic and more severe forms of disease that typically occur in adulthood [15-18]. Recently, two reviews analysed published data on anti-HAV seroprevalence in countries of North and West Africa and Middle East and reported a gradual shift in the age of infection from early childhood to late childhood or adulthood, indicating a shift towards intermediate endemicity in these areas [19,20].

In Tunisia, HAV epidemiology has changed from a high to an intermediate endemicity pattern, particularly in urban areas [11]. Improvement of hygiene and socioeconomic conditions has undoubtedly contributed to this epidemiologic shift. However, seroprevalence rates are still more elevated than those reported in European countries. Child infection rates remain high, with differences between urban and rural settings, depending on the development of the considered areas [11,12,21]. Lower anti-HAV prevalences were found in coastal regions, as compared to the rest of the country: this difference may be due to the higher socioeconomic level of the coastal populations [22]. Although HAV incidence has declined over the past decades, in Tunisia many outbreaks continue to occur.

Based on nucleotide sequence analysis, human HAV is classified in 3 genotypes (I, II and III) and sub-classified in 6 sub-genotypes (IA, IB, IIA, IIB, IIIA, IIIB). Molecular characterization of HAV strains from Tunisian patients showed a clear predominance of sub-genotype IA (about 98%), compared to sub-genotype IB (2%); no II and III genotypes were found [23,24].

In the present study, samples collected in different towns in Tunisia during 2008–2013 from patients with acute hepatitis A were analysed by nested RT-PCR and sequencing to characterize HAV isolates. Tunisian HAV sequences, both from the present study and downloaded from Genbank and overall including cases from 2001 to 2013, were analysed by phylogenetic and migration pattern analyses to investigate, for the first time, the viral gene flow of HAV in Tunisian towns.

## Results

### Virological characterization of HAV isolates

**Figure 1:**
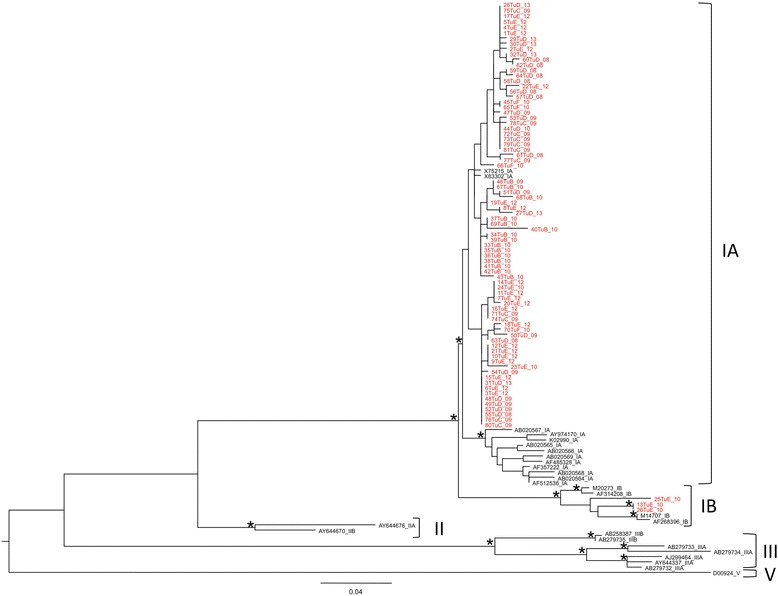
**Maximum likelihood phylogenetic analysis of HAV sequences (VP1/2A region).** Phylogenetic tree of 81 HAV sequences from Sousse, Mahdia, Monastir, Tunis and Kairouan and 27 reference sequences (genotype I, II, III and V) downloaded from GenBank. The 81 sequences from Tunisia are highlighted in red; their name includes, after a progressive number (1 to 81) followed by “Tu”, a capital letter indicating the town from which the sample was collected (A: Sfax; B: Sousse; C: Mahdia; D: Monastir; E: Tunis; F: Kairouan) and, at the end of the name, the sampling year (e.g. _09 stands for 2009, _10 stands for 2010, and so on). See Methods for further details. The 27 reference sequences are reported by their Accession number followed by the genotype they belong to. Branch lenghts were estimated with the best fitting nucleotide substitution model (TrN + I + G) according to a hierarchical likelihood ratio test [38], the scale bar at the bottom indicates 0.04 nucleotide substitutions per site. One *along a branch represents significant statistical support for the clade subtending that branch (p < 0.001 in the zero-branch-lenght test and bootstrap support >75%).

HAV RNA tested positive by nested RT-PCR in 81 of 190 analysed samples collected in different Tunisian towns during 2008–2013. The features of the PCR positive group were not dissimilar from those of the total group of patients included in the study (described in the Methods section): the mean age of the 81 patients was 9.7 years, the median age was 8.0 years and the age range was 2–60 years; 45 patients were males (55.6%) and 36 females (44.4%). Sequencing of the 81 PCR products followed by phylogenetic analysis with genotype reference sequences showed that most isolates (78/81, 96.3%) were genotype IA, while only three sequences, all from Tunis patients, were genotype IB (3/81, 3.7%) (Figure 1).

Pair-wise comparisons of the sequenced VP1/2A region of isolated genotype IA strains revealed a genetic identity level ranging from 94.8% to 100%. The same identity range was obtained by including in the analysis Tunisian strains downloaded from Genbank.

**Figure 2:**
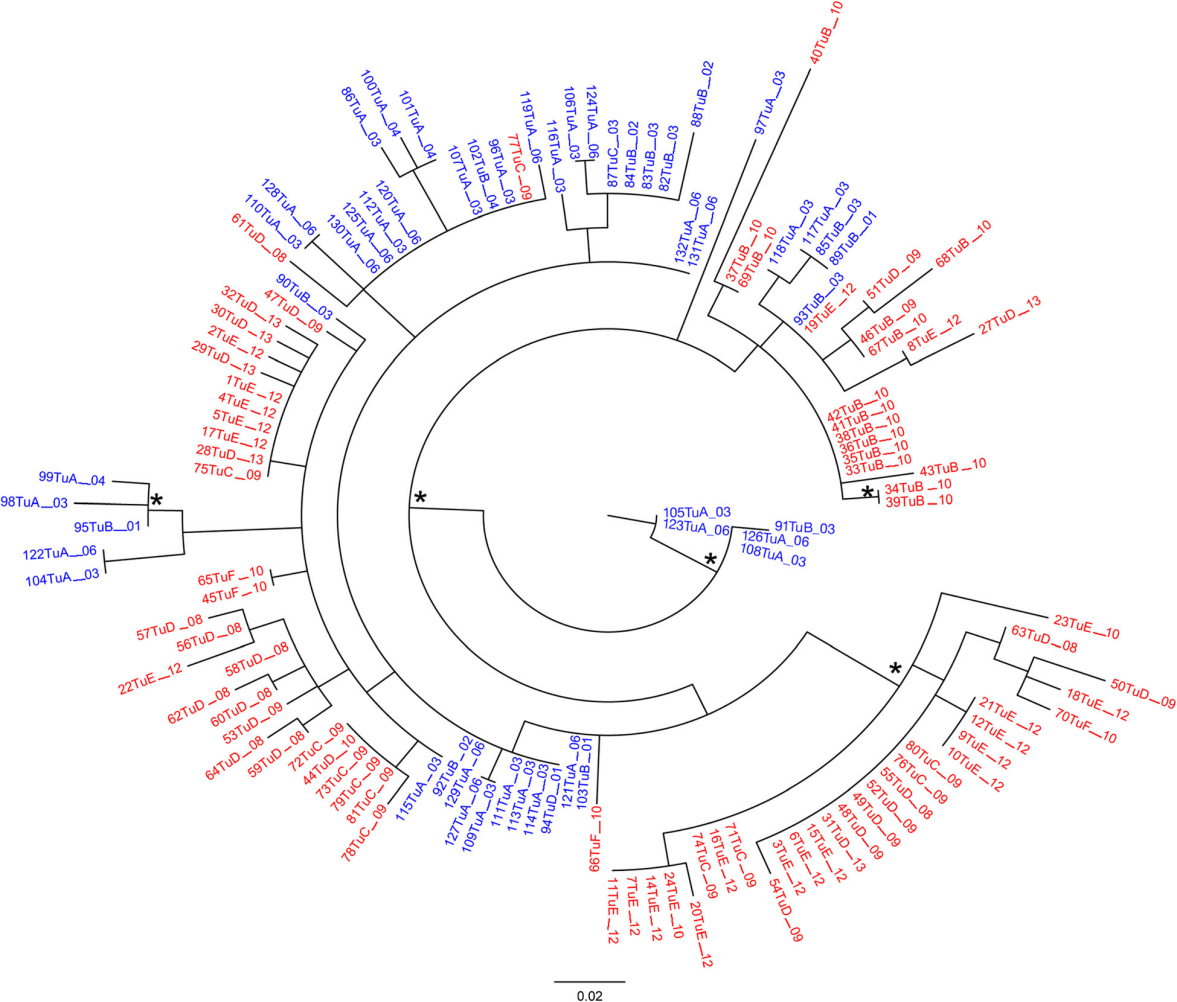
**Maximum likelihood phylogenetic analysis of HAV genotype IA sequences (VP1/2A region).** Phylogenetic tree of 129 genotype IA Tunisian HAV sequences from six Tunisian towns (78 new sequences from the present study and 51 reference sequences from GenBank). Branch lenghts were estimated with the best fitting nucleotide substitution model (HKY + G) according to a hierarchical likelihood ratio test [38], the scale bar at the bottom indicates 0.02 nucleotide substitutions per site. One *along a branch represents significant statistical support for the clade subtending that branch (p < 0.001 in the zero-branch-lenght test and bootstrap support >75%). The 78 sequences from the present study are highlighted in red, the 51 reference sequences in blue. Sequence names of new and reference sequences include town and year of sample collection (see the “Sequence datasets” section in Methods).

Figure 2 shows the phylogenetic tree of 129 genotype IA isolates from six Tunisian towns. The presence of several distinct clusters in the phylogenetic tree revealed the circulation of different genotype IA strains.

### Migration pattern of HAV Tunisian IA genotype

**Figure 3:**
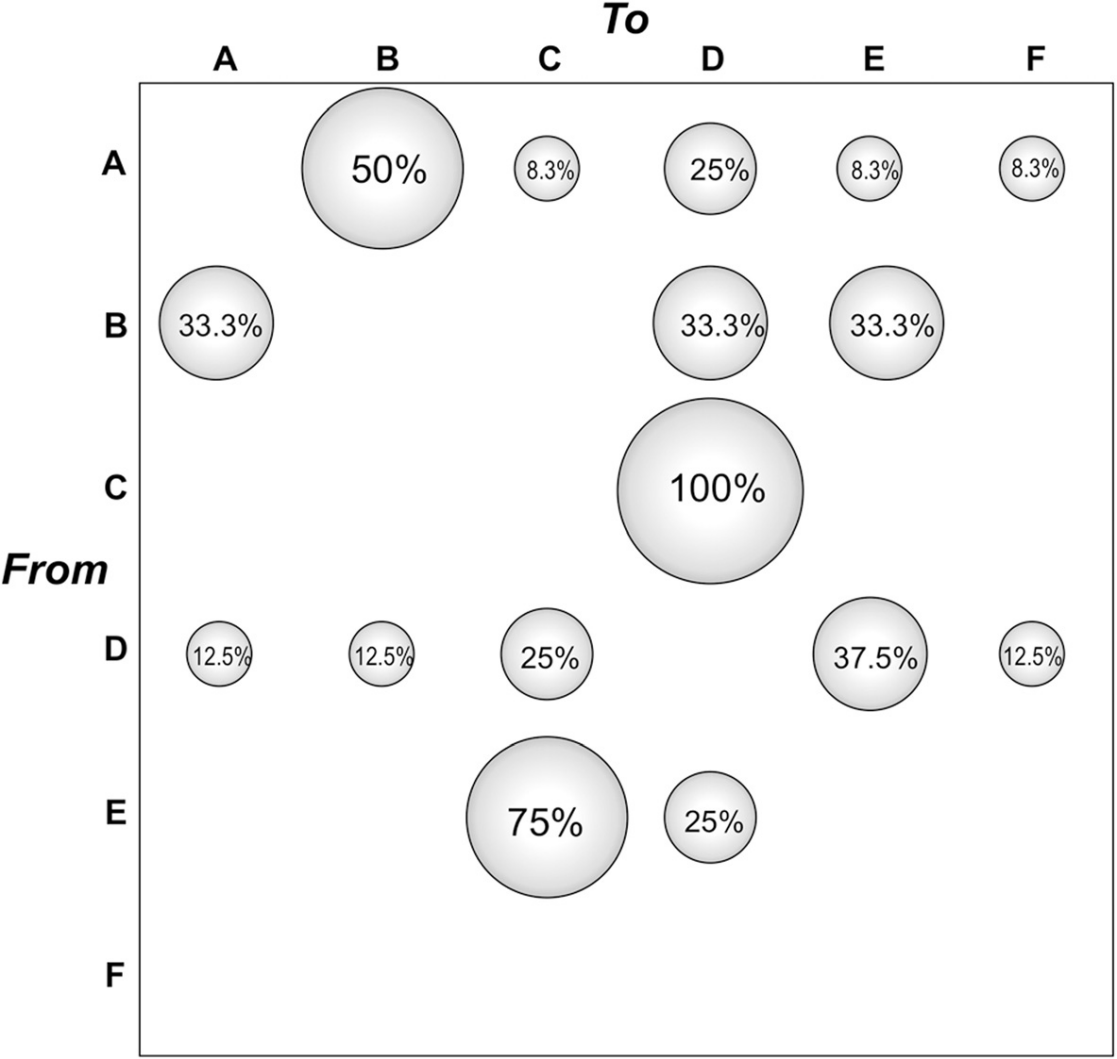
**Migration pattern of HAV genotype IA circulation in Tunisia.** The bubblegram shows the frequency of gene flow (migrations) in Tunisia to/from different geographic areas (towns). The surface of each circle is proportional to the percentage of observed migrations in the ML genealogy. Migrations were inferred with a modified version of the Slatkin and Maddison algorithm [25,26] for the HAV subtype IA from the maximum likelihood inferred genealogies given in Figure 2. A: Sfax; B: Sousse; C: Mahdia; D: Monastir; E: Tunis; F: Kairouan.

The gene flow (migration) of HAV genotype IA among 6 major towns in Tunisia (A: Sfax, B: Sousse, C: Mahdia, D: Monastir, E: Tunis and F: Kairouan) was investigated with a modified version of the Slatkin and Maddison method [25]. After superimposing the town of origin of the Tunisian sequences on the tip branches of the ML genealogy, we inferred the town of origin of each ancestral node (i.e. ancestral sequence) using the maximum parsimony algorithm. The null hypothesis of panmixia (i.e. no population subdivision or complete intermixing of sequences from different geographical areas) was tested using a bubblegram. The migration flow among the six towns was then estimated as observed migration in the genealogy (Figure 3). The null hypothesis of panmixia was rejected by the randomization test (p = 0.0001) for all the towns in the bubblegram [26].

**Figure 4:**
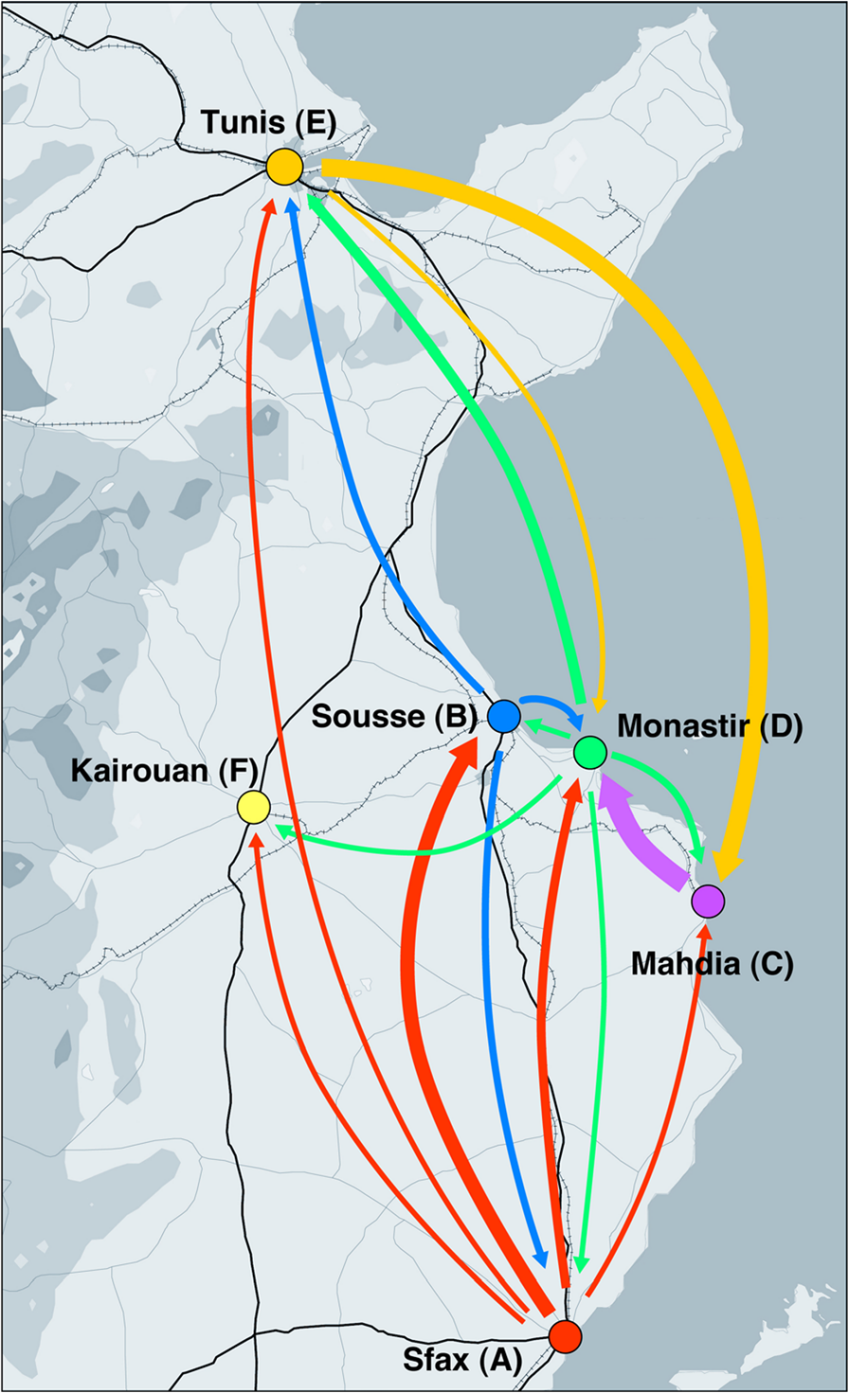
**Detailed mapping of HAV IA gene flow (coloured arrows) among some North-Central Tunisian towns.** The line thickness of the arrows is roughly proportional to the percentage of observed migrations.

As shown by the bubblegram in Figure 3, the viral gene flow from each of two towns (Sfax and Monastir) appears to be directed to all the others: the flow from Sfax is directed mainly to Sousse (50%) and to a less extent to Monastir (25%), Mahdia (8.3%), Tunis (8.3%) and Kairouan (8.3%); the flow from Monastir appears more equally distributed among the five towns (Sfax 12.5%, Sousse 12.5%, Mahdia 25%, Tunis 37.5% and Kairouan 12.5%) (Figure 3). On the other hand, the viral gene flow from Sousse was equally distributed towards three of the five towns (Sfax, Monastir and Tunis, 33.3% each), while Tunis showed viral gene flow towards only two towns (mainly to Mahdia, 75%; to a less extent to Monastir, 25%); finally, the flow from Mahdia was directed exclusively to one town (Monastir, 100%). No viral gene flow from Kairouan to the other towns was observed. Overall, a complex viral gene flow among the considered Tunisian towns emerges, as schematically summarised in Figure 4.

## Discussion

Although the incidence of infection in Tunisia has declined over the last years, HAV outbreaks continue to occur [11,24]. Person-to-person transmission and consumption of contaminated water are main risk factors for the Tunisian population [11,24,27]. Sea food consumption, especially raw shellfish due to their bio-concentrator ability, could also play a potential role in transmission, because of residual HAV contamination of effluents from waste water treatment plants [2,28,29]. Moreover, climatic conditions, soil leaching caused by heavy winter rainfalls, and the use of unsanitised sludge as crop fertiliser are further factors that may explain the intensity of the epidemics during the winter [30].

Most Tunisian sequences reported in previous studies (spanning the 329 nt region sequenced in the present study) were collected from two towns (Sfax and Sousse) and the most recent of them date back to 2006 [23,24]. In those studies, the authors showed circulation of different HAV IA strains, some of them genetically related to strains isolated in other Mediterranean countries.

The present study is the first large-scale molecular study analysing clinical sequences from six Tunisian towns, some of them never sampled in the past; in addition, it represents an updated overview of the strains circulating in Tunisia. The great majority of isolates (78/81, 96.7%) proved to be genotype IA, in agreement with previous studies that reported about 98% HAV IA strains both in clinical and in environmental samples in Tunisia [23,24,29-31].

The circulation of different genotype IA strains is supported by the presence of several distinct clusters in the phylogenetic tree, as also reported in other Tunisian studies (Figure 2). This pattern is in accordance with the sampling mode (samples were collected randomly during multiple years, not in an outbreak) and confirms that different strains co-circulate in Tunisia [23].

Ten clinical strains characterized in the present study proved to be identical to the majority of IA strains found in urban wastewaters in Tunisia [29]; in particular, a strain detected in a patient from Tunis in 2012 was identical to the most prevalent environmental strain, found in 42 wastewater samples [29]. Importantly, the above ten strains were characterized from samples recently collected (2008 to 2013) from patients in Tunisian regions not previously investigated: this finding confirms current extensive circulation of clinical strains in the environment, suggesting the importance of implementing a clinical-environmental surveillance system, to monitor the efficacy of preventive and control measures.

In the present study either serum or stool was sampled, which can raise concerns for possible introduction of a bias in the gene migration pattern analysis due to slight sequence differences between virions in serum and in stool. Actually, this possibility is unlikely because a recent study showed that identical HAV sequences are obtained from different clinical samples (serum, stool, saliva and urine) of the same patient [32].

Migration analysis showed a complex virus gene flow among the towns considered in the present study. However, examination of Figure 3 suggests (1) an overall South-to-North major flow from Sfax to Sousse and, to a less extent, to Monastir, and from these two latter towns to Tunis, and (2) an overall North-to-South major flow from Tunis to Mahdia and then (3) from Mahdia again South-to-North to Monastir; a complex pattern of minor flows is also visible. Multiple factors are likely involved in determining the observed flows: demographic (population size and movement), climatic (the floods frequently occurring in the raining season), economic (food trade); the underlying infrastructures may also play a role (such as the lack of an extensive and widespread distribution of sewage systems). Interactions between these factors, as in the case of floods and poor sewage systems, may determine the optimal conditions for spread of an enterically transmitted and highly environmental resistant agent such as HAV.

## Conclusions

In conclusion, this is the first study describing by a migration pattern approach the circulation of HAV in North-Central Tunisia towns. Currently, genotype IA predominates widely, with several newly and previously identified strains. The viral gene flow analysis of the present study, coupled to a wider sampling, can be useful to investigate the factors underlying the spread of HAV infections in Tunisia and, thus, to implement appropriate preventing measures.

## Methods

### Patients and samples

Serum or stool samples were collected from 190 patients with symptoms of acute hepatitis as part of standard care: serum samples from 145 patients hospitalized in Tunis (44 patients, Hospital Charles Nicolle; Hospital Rabta; Pasteur Institute), Sousse (26 patients, Hospital Farhat Hached), Monastir (61 patients, Hospital Fattouma Bourguiba), Kairouan (14 patients, Hospital Ibnou El Jazzar) in 2008–2013; stool samples from 45 hospitalized patients by the Regional Direction of Public Health in Mahdia in a one year survey during 2009. The study was in compliance with the Helsinki Declaration and was approved by the Ethic Committee of Hôpital Universitaire Fattouma, Monastir (President of the Ethic Committee: Prof. Fekri Abroug).

The mean age of the 190 patients was 12.4 years, the median age was 9 years and the age range was 2–66 years; 109 patients were males (57%) and 81 females (43%). Informed consent for characterization of virus in samples was obtained before patient discharge. The study conformed to the Declaration of Helsinki. All cases were diagnosed locally as HAV infections on the basis of serum antibody detection by a commercial test (Abbott Architect HAVAB-M, Abbott Diagnostics, Abbott Park, Illinois). Virological analysis of samples was carried out by HAV RNA detection and sequencing at the Viral Hepatitis Unit of the Italian National Institute of Health in Rome.

### Nested RT-PCR and Sequencing

Viral RNA extraction from all samples (sera and stools) was carried out by the QIAamp Viral RNA Mini Kit (Qiagen GmbH, Hilden, Germany). For serum, a 140 μl volume was extracted according to the manufacturer’s protocol. For stools, a modified protocol downloaded from the QIAGEN site, including an additional preliminary step, was used [33]. In the additional preliminary step, 0.2 ml of stool was carefully suspended in 2 ml of 0.89% NaCl. The suspension was clarified by centrifugation for 20 min at 4,000 × g. The supernatant was filtered using a 0.22 μm syringe filter (Millipore), and 140 μL of eluate was then used for HAV RNA extraction. The remainder of the procedure is common to stool and serum. The RNA was recovered in 60 μL elution buffer and a 394 base pairs (bp) DNA fragment spanning the VP1/2A junction of HAV genome was amplified by nested RT-PCR as previously reported, with minor modifications [34]. In order to increase the sensitivity, a nested PCR approach was preferred. In brief, 10 μL extracted RNA was reverse transcribed with 2.5 μM random hexamers (final concentration) and 200 U of Moloney Murine Leukemia Virus reverse transcriptase (Invitrogen, by Life Technologies, Carlsbad, CA) in a final volume of 20 μL at 50°C for 1 hr, followed by 15 min at 70°C. One-half cDNA volume (10 μL) was used as template in the first PCR round. After denaturation for 2 min at 94°C, DNA was amplified in a 50 μL final volume for 30 cycles at 94°C for 30 sec, 40°C for 30 sec, and 72°C for 1 min, followed by a final extension step at 72°C for 7 min. Reaction mixture was as follows: 1X PCR buffer, 1.5 mM MgCl_2_, 0.2 mM each dNTP, 1 μM each primer, with 1 U of Platinum Taq DNA polymerase per 50 μL reaction (Invitrogen, by Life Technologies, Carlsbad, CA).

In order to improve the ability to amplify any HAV genotype, a modified degenerated version of previously published primers was used [33]. Primers in the first round of PCR were: sense +2870 (5′-GACAGATTCYACATTTGGATTGGT-3′) and antisense −3381 (5′-CCATYTCAAGAGTCCACACACT-3′), where Y represents C or T. Nested PCR was carried out with 0.5 ul of the first round PCR product as template for 35 cycles, with the same reaction and cycling conditions of the first PCR. Inner primers were: sense +2896 (5′-CTATTCAGATTGCAAATTAYAAT-3′) and antisense −3289 (5′-AAYTTCATYATTTCATGCTCCT-3′). PCR products (10 ul) were loaded on a 2% agarose gel, electrophoresed and stained with ethidium bromide to visualize bands of expected size (394 bp).

PCR products were sequenced using the GenomeLab DTCS Quick Start KiT (Beckman Coulter, Fullerton, CA) following the manufacturer’s instructions, with the same primers used in nested PCR (sense +2896 and antisense −3289, see above) and an automated DNA sequencer (Beckman Coulter, Fullerton, CA). After editing and alignment, a common 329 nt region was available for analysis.

### Sequence datasets

The 81 Tunisian HAV sequences from the present study have been deposited in GenBank [GenBank:KP091352 to KP091432]. Their sequence names, reported in Figures 1 and 2, were coded as follows: a progressive number (1 to 81), followed by “Tu” and a capital letter (A, B, C, D, E or F) indicating the sampling town (A: Sfax; B: Sousse; C: Mahdia; D: Monastir; E: Tunis; F: Kairouan) and, finally, the sampling year preceded by “_”. For instance, 27TuD_13 indicates the Tunisian sequence 27, sampled in Monastir in 2013.

Two sequence datasets were built. The first one was built to determine the genotype of each isolate and included all the 81 newly obtained sequences plus 27 reference sequences from several countries representative of genotypes I, II, III (human strains) and V (a simian strain), downloaded from the GenBank database via the National Center for Biotechnology Information (NCBI) site [35]. The list of the Accession Number of the 27 reference sequences retrieved from GenBank follows. Genotype IA: AF357222; AB020565; AB020564; AB020567; AB020566; AB020568); AB020569; AF485328; AF512536; X75215; X83302; AY974170; K02990. Genotype IB: M14707; M20273; AF268396; AF314208. Genotype IIA: AY644676. Genotype IIB: AY644670. Genotype IIIA: AJ299464; AY644337; AB279732; AB279733; AB279734. Genotype IIIB: AB258387; AB279735. Genotype V (simian virus): D00924.

The second dataset was built to perform phylogenetic analysis and viral gene flow exclusively on genotype IA Tunisian sequences. This dataset included 78 new sequences from the present study (identified as genotype IA among the above 81 new sequences) and 51 Tunisian sequences downloaded from GenBank. These latter sequences were selected according to the following inclusion criteria, based on information available from sequence annotations or from published studies: (1) known sampling town; (2) known sampling year. Their names were coded exactly as the newly obtained sequences (see above): a number (82 to 132, to continue the progressive numbering) followed by “Tu”, then a capital letter (A, B, C, D, E or F) indicating the sampling town and, finally, the sampling year preceded by “_”. The list of the 51 downloaded Tunisian sequences collected in Sfax, Sousse, Mahdia and Monastir from 2001 to 2006 with matched Accession Number (in parentheses) follows: 82TuB_03 (AY875650); 83TuB_03 (AY875651); 84TuB_02 (AY875652); 85TuB_03 (AY875653); 86TuA_03 (AY875654); 87TuC_03 (AY875655); 88TuB_02 (AY875656); 89TuB_01 (AY875657); 90TuB_03 (AY875659); 91TuB_03 (AY875660); 92TuB_02 (AY875661); 93TuB_03 (AY875662); 94TuD_01 (AY875663); 95TuB_01 (AY875664); 96TuA_03 (AY875665); 97TuA_03 (AY875666); 98TuA_03 (AY875667); 99TuA_04 (AY875668); 100TuA_04 (AY875669); 101TuA_04 (AY875670); 102TuB_04 (AY875671); 103TuB_01 (AY875672); 104TuA_03 (DQ380510); 105TuA_03 (DQ380511); 106TuA_03 (DQ380512); 107TuA_03 (DQ380513); 108TuA_03 (DQ380514); 109TuA_03 (DQ380515); 110TuA_03 (DQ380516); 111TuA_03 (DQ380517); 112TuA_03 (DQ380518); 113TuA_03 (DQ380519); 114TuA_03 (DQ380520); 115TuA_03 (DQ380521); 116TuA_03 (DQ380522); 117TuA_03 (DQ380523); 118TuA_03 (DQ380524); 119TuA_06 (HM011106); 120TuA_06 (HM011107); 121TuA_06 (HM011108); 122TuA_06 (HM011109); 123TuA_06 (HM011110); 124TuA_06 (HM011111); 125TuA_06 (HM011112); 126TuA_06 (HM011113); 127TuA_06 (HM011114); 128TuA_06 (HM011115); 129TuA_06 (HM011116); 130TuA_06 (HM011117); 131TuA_06 (HM011118); 132TuA_06 (HM011119).

Sequences were aligned with the Clustal algorithm [36], then edited manually. The Tunisian genotype IA dataset was subjected to a preliminary likelihood mapping analysis to assess if a suitable phylogenetic signal could be detected, as previously described [37,38].

### Phylogenetic analysis

The best fitting nucleotide substitution model was tested with a hierarchical likelihood ratio test following the strategy described by Swofford and Sullivan [38], using a neighbour-joining (NJ) based-tree with LogDet corrected distances. Maximum likelihood (ML) trees were then inferred with the selected model and ML-estimated substitution parameters. The heuristic search for the ML tree was performed using a NJ tree as starting tree and the TBR branch-swapping algorithm. NJ trees were also estimated using pair-wise distances inferred by ML with the best fitting nucleotide substitution model. Calculations were performed with PAUP 4.0b10 [39]. Statistical support for internal branches in the NJ trees was obtained by bootstrapping (1000 replicates) and with the ML-based zero branch length test for the ML trees [38]. Trees were rooted by ML rooting by selecting the rooted tree with the best likelihood under the molecular clock constraint (taking into account the different sampling times of the taxa) [40,41].

### Migration pattern

The MacClade version 4 program (Sinauer Associates, Sunderland, MA) was used to test viral gene in/out flow among distinct HAV subpopulations within different geographic regions and towns in Tunisia (from North to South: Tunis, Sousse, Monastir, Kairouan, Mahdia and Sfax), using a modified version of the Slatkin and Maddison test as described below [25].

A one-character data matrix is obtained from the original data set by assigning to each taxon in the tree a one-letter code indicating its city of origin. Then, the putative origin of each ancestral sequence (i.e. internal node) in the tree is inferred by finding the most parsimonious reconstruction (MPR) of the ancestral character. The final tree-length, i.e. the number of observed migrations in the genealogy, can easily be computed and compared to the tree-length distribution of 10,000 trees obtained by random joining-splitting.

Observed genealogies significantly shorter than random trees indicate the presence of subdivided populations with restricted gene flow [25]. Specific migrations among different towns (character states) were traced with the State changes and stasis tool (MacClade software), which counts the number of changes in a tree for each pair-wise character state. When multiple MPRs were present (as in our data set), the algorithm calculated the average migration count over all possible MPRs for each pair. The resulting pair-wise migration matrix was then normalized, and a randomization test with 10,000 matrices obtained from 10,000 random trees (by random joining-splitting of the original tree) was performed to assess the statistical significance of the observed migration counts.

### Consent

Written informed consent was obtained from the patients, or their parents in the case of minors, for participation in the study.
